# Emotion in Career-Related Transitions of Young Adult Immigrants: A Contextual Action Theory Perspective

**DOI:** 10.1177/08948453251382176

**Published:** 2025-10-16

**Authors:** Richard A. Young, José F. Domene, Yan Liu, Kesha Pradhan, L. Alejandra Botia, Eugene Chi

**Affiliations:** 1 Department of Educational and Counselling Psychology, and Special Education, 8166 The University of British Columbia, Vancouver, BC, Canada; 2 Werklund School of Education, 152803 University of Calgary, Surrey, BC, Canada; 3 Department of Psychology, 6339 Carleton University, Ottawa, ON, Canada

**Keywords:** emotion, career, immigrant, transition to adulthood, cases

## Abstract

Emotions are critical intrapsychic and relational processes in the meaningful actions of people. In this study, we illustrate the role of emotion in the career-related actions and projects of two dyads of young adults who participated in a program to support their transition to adulthood and to living in a new country. Contextual action theory (CAT) provided the framework to understand emotion both as an intrapsychic and relational process. The action-project qualitative method was used to collect and analyze data. Data for each dyad included joint conversations, video recall of emotions and cognitions during the joint conversations, and the identification and monitoring of the dyad’s joint transition project. The cases demonstrate how emotions emerge during key career development transitions and are addressed and supported in the actions between people. They further highlight how our engagement with others can help or hinder individuals’ processes through these transitions, including the role of emotional regulation. The illustrations of emotional processes in these dyads point to the salience of emotion in career construction and counseling.

The transition process for young adult immigrants to Canada and other countries is complex, broadly career related, and emotion inducing (e.g., Record-Lemon et al., 2021). For example, stress, among several emotional concerns, has been documented for both international students and young adult immigrants (e.g., Sinacore & Lerner, 2013; Smith & Khawaja, 2011; Wong et al., 2017). Nota and colleagues (2014) have noted that migrants are among those with significant career development needs. Typically, immigrants are engaged in a range of emotions that serve to construct their transition-related actions.

Transition has been a significant topic in psychological research generally and the career literature specifically (e.g., Meerkins et al., 2021). Broadly it refers to a change from one position or state of life to another. Most notable in the career field is the change from student to fulltime employee, but other career transitions are also significant, for example, the transition from being employed to retirement (Froidevaux et al., 2016) or work transitions (Reitz et al., 2022), not the least significant and challenging is the complex transition to both a new country and adulthood. This complexity was identified in Popadiuk and Arthur’s (2021) review of the literature on the transition to work for international students, which attests to the complexity of this process ranging from identity processes to building social capital. The transitional tasks for young or emerging adults include entering the workforce, finding employment, acquiring post-secondary education, and starting a family (Sabella et al., 2020; Sulimani-Aidan, 2024). An extensive literature provides evidence of the emotional engagement in the school-to-work transition entails for young people (e.g., Upadyaya et al., 2021; Vignoli et al., 2020). Suárez-Orozco and colleagues (2018) highlighted the complexity of transition by identifying four level of influence, namely, intraindividual, microsystem, political, and global.

The recent research on transitions has pointed to several factors that reflect the complexity of transition. For example, McLaughlin (2023) pointed to the tension between explanations based on structural, societal expectations and more intersectional perspectives in which the “fluid, uncertain, and dynamic” characteristics of transition are reflected. Dougherty (2022) called for moving away from the discourse of social mobility as the foundational concept in transition research. Personal goals may be one way to address this intersectionality as they have been found to guide transition behavior (e.g., Dietrich et al., 2012). In this paper, we assume that identifying a single outcome for the transition of young people moving from education to employment is not desirable. Rather our view is that transition is a complex, multifaceted process. Indeed, access to the variety of actions in which people engage in making a transition can reveal the complexity and diversity of this process of which emotion is a part.

Twenty-five years ago, Kidd (1998) noted the absence of a clear place for emotion in career research and practice. However, recent research has reflected an increased attention being paid to the emotion related to a range of life transitions, for example, transitions to parenting (Cao et al., 2022), online teaching (Naylor & Nyanjom, 2021), secondary school (De Moor et al., 2023), career (Vignoli et al., 2020), and retirement (Horn et al., 2021). Indeed, Watson (2015) proposed emotion-focused therapy as an alternative to career counseling, suggesting that “[w]orking with emotional processes … is vital to assist people to make good and satisfying decisions” (p. 146). Of course, emotion itself is a complex construct. It refers broadly to the adaptive response of humans in which they appraise the situation or context and engage in subsequent action (Scherer & Moors, 2019). There are also a variety of specific emotion processes and terms that have been identified and used in the literature. These include emotional intelligence, emotion regulation, affect, and mood. In this article, our purpose is to illustrate the emotional processes between young adults as they engage in transition-related projects related to their simultaneous transitions to a new country and career. The aim of this paper is to illustrate through two case examples, the intrapersonal and interpersonal emotions that young adult newcomers to Canada experience as they engage in joint transition-oriented projects.

## Theoretical Framework

Given the challenges of career-related immigrant transitions and the place that interpersonal support plays in that process, it is imperative that greater attention be paid to these processes and the role of emotion in them. One conceptual approach is provided by contextual action theory (CAT, Young, Domene, Valach, & Socholotiuk, 2021; Young & Valach, 2008), which is a complex, relationally oriented framework for understanding human action and, over the long term, career. CAT has been recognized as a conceptual approach to understanding career (Brown & Associates, 2002; Skorikov & Patton, 2007) and has been applied to career counseling practice (e.g., McMahon, 2017; Savickas & Walsh, 1996) for over two decades.

CAT views occupational and other careers as constructed through a series of goal-directed processes and actions. This constructionist perspective facilitates the understanding of career-related processes, including agency, meaning, and the joint actions between individuals. As a framework, CAT and the associated action-project research method have been used to describe a variety of career-related processes, for example, young couples’ transition to work (Domene et al., 2012), the transition to high school (Marshall et al., 2014), the transition to high school for young persons with a disability (Marshall et al., 2019), and early sport specialization (Wall et al., 2020). As a framework, CAT highlights the place of action with others as well as the role of emotion in those actions.

CAT responds to the call for increased attention to the varied aspects of transition by addressing the relevant actions and projects undertaken by people engaged in the transition process. For example, Sullivan and Arris (2021) point to the need for career research to address the role of social networks and multiple identities in career research. CAT focuses on how transition processes are embodied and engaged jointly in daily life. This process is bi-directional. Even as the person engages with others to realize their goals, they also reassess their own goals based on their actions with others.

### Emotion and Contextual Action Theory

The primary focus of contextual action theory is that much of human behavior is goal-directed and intentional. Emotion plays an important role in significant human action. For example, feelings of failure can lead a person to abandon a post-secondary educational program. Similarly, family support can generate confidence in aspiring to a career as a medical doctor. Contextual action theory holds that emotion can facilitate or impede a person’s actions. In line with Elfenbein (2023) and Frijda (1986), CAT maintains that emotions not only communicate information about internal states, but they also influence actions between people. The more critical the action, the greater the significance of the emotion. In addition to its energizing function, emotion has a steering and regulating function. Importantly, contextual action theory sees human action as complex and socially constructed that includes a host of manifest, cognitive/emotional, and social processes.

Contextual action theory also focuses on the way in which actions involve others in significant ways (Young, Domene, & Valach, 2015). In particular, the theory focuses on the joint actions that individuals undertake with others. From this standpoint, the transition projects of young adult immigrants are often constructed in relation to and with others in their lives. Given the challenge of transitions and the importance of others in the transition process, one would expect that emotions play a significant role in them.

In this paper, we illustrate the role of emotion in the transition projects of two young adult newcomer dyads. Here, dyad refers to two young adult newcomers to Canada who chose to participate in a supportive counseling intervention that focused on the goal-directed actions and projects of young adult immigrants to Canada. Various aspects of the study have been reported elsewhere (Young, Domene, Botia, et al., 2021; Young et al., 2020, 2022, 2024). This article focuses on the emotion processes in two dyads whose transition projects were broadly related to their educational/occupational careers.

## Method

The larger study from which the following case illustrations were extracted was an exploratory study that described the joint transition-oriented projects that young adult newcomers to Canada participated in together. The two cases reprinted here were chosen as examples of the role of emotion in career-oriented transition.

### Larger Study Method

The study from which these two cases were taken used a collective case study design (Stake, 2000). Instrumental case study reports were used to gain a broad understanding of the transition-related goal-directed projects of young newcomers and how these projects can be supported. The research design was informed by the action-project method, a relational, social constructionist qualitative method grounded in the assumptions of CAT (Young et al., 2002, 2021). Qualitative data were collected as participants engaged in a transition support program. Participants were recruited through notices on social media and distributed to university cultural groups.

The supportive intervention in which these dyads were engaged occurred over approximately a 3-month period. It was based on the action-project method (APM, Young et al., 2005). The purpose of the intervention was to identify and support the joint goal-directed projects of the dyad participants related to their transition to adulthood and a new country. The intervention consisted of three in-person meetings of the dyad with two counselors and separate follow-up telephone calls to each participant (for a full description of the intervention, see Young et al., 2024).

In the first meeting, the counselors facilitated a conversation between the client dyad relevant to the issue for which they sought support. In a video-playback session immediately following the dyadic conversation, clients separately reviewed the recorded conversation with the counselor, who invited the client to share their thoughts and feelings as the dyadic conversation progressed. The main purpose of the video playback was to activate the clients’ subjective processes, including emotions, critical to their actions. These processes may or may not have been readily apparent in the manifest behaviors in their conversation. In the second meeting, written narratives of the initial conversation were presented, a specific joint transition project that emerged from the first conversation was identified, and suggestions about how to further the project were made. During this meeting, the counselors worked collaboratively with the clients to ensure that their transition project was accurately captured and to discuss the suitability of the suggestions. The counselors then connected with clients separately through telephone monitoring calls every 2 weeks over 3 months. The purpose of the telephone monitoring was to reinforce the clients’ engagement with each other in their transition project. Finally, the third and final in-person meeting followed the same procedure as the first meeting, with a joint conversation between the dyad followed by the video playback. The session allowed for project integration and progress to be addressed.

In APM, each case analysis aims to identify and describe the joint transition-oriented project in which the dyad participants engaged. A preliminary analysis was conducted following the initial session and a more thorough analysis was conducted after all stages of data collection had been completed (Young et al., 2005). This analysis was qualitative and grounded in the theoretical tenets of CAT. It relied on hermeneutic cycling between the whole and the parts of the extensive data set, that is, interview transcripts, video recordings, video-playback descriptions, and telephone monitoring logs. It also relied on consensus-based decision-making among the team of researchers who discussed multiple possibilities until agreement was reached as to the most plausible interpretation. Analysis of the transcribed data occurred line-by-line, wherein the researchers identified elements, functional steps, and goals for each minute of the conversation. The analysis procedure used in these cases illustrated in this article is described in greater detail elsewhere (Young, Domene, Liu, et al., 2024, also see Wall et al., 2016). The action-project method is fully described in Young, Domene, Valach and colleagues (2021).

During the preliminary analysis, particular attention is paid to the perspectives and levels of action present in the joint conversation and self-confrontation. This in-depth analysis reveals how participants interact with each other in conversation and provides an indication of the ways they may act in pursing their social inclusion projects. For example, in a study of couples attempting to transition into the workforce, some couples had a partner who tended to initiate conversational topics and ask questions or a partner whose actions were mostly responsive/reactive in nature. In contrast, other couples had a more egalitarian interactional style (Domene et al., 2012). The patterns of action that emerged from this in-depth analysis, combined with additional information obtained from the warm-up and self-confrontation, also revealed the specific social inclusion projects that were meaningful to participants at that time.

The final analysis also focused on participants’ actions, along with uncovering patterns of action taken towards participants’ projects, the progress they made, and how their goals, projects, and relationship may have changed over time (Valach et al., 2002; Young et al., 2005). At this stage, information from the monitoring period and final interview were combined with the results of the preliminary analysis. The result was a rich description of the projects and joint actions of participants, encompassing their behavior, thoughts and emotions, and the meanings they constructed as they acted to achieve goals.

### Selection of Two Illustrative Cases

From the 15 cases who participated in the larger research study, two illustrative cases were purposively chosen because career and emotion were explicit in them. The data collection method allowed the authors to examine the place of emotion in these cases with a degree of specificity often not available in other cases or data sets.

### Rigor and Trustworthiness of Qualitative Procedures

Young and colleagues (2024) described the rigor and trustworthiness of the qualitative research procedures used in the larger study from which the cases presented here are extracted. Briefly these cases included collecting data that were both retrospective and prospective, grounding the analysis in both the data and the guiding theory, the data analysis was triangulated with data from various sources (interview, video-recorded observation of participant-counselor interactions, counselor notes from telephone contacts). In addition, during the second meeting, participants had the opportunity to review the data analysis of the first data collection meeting.

### Researcher Reflexivity

The principal investigators had previous experience with the conceptual framework and method used in this study. They included both immigrants and non-immigrants to Canada. Two self-identified as men and one as a woman. Three counselors involved in this supportive counseling intervention were advanced graduate students in counseling psychology who had been trained broadly in the action-project method and specifically for the supportive intervention used in these cases. Two counselors self-identified as women and one as man. One counselor was an immigrant to Canada.

## Case Illustrations

### BA and EL

BA and EL (note all initials throughout are pseudonyms to protect anonymity) were a couple who emigrated from the same region of their home country. The goal of their project was to improve their communication around career issues and to facilitate their career exploration as they transitioned to life in Canada. This project was part of their broader journey of maintaining a relationship while being international students in the final year of their undergraduate degrees. BA and EL provide an example of how anxieties about establishing a career in a foreign country can manifest in emerging adults. This transitional stage of development was meaningful for them. They were leaving their roles as students and establishing themselves as independent adults, resulting in pressure to “figure [it] out” before completing their degrees. While both were experiencing a similar transition, their individual socio-historical contexts influenced the meaning-making process, the emotions experienced, and actions they engaged in. Emotion can be observed as BA and EL worked together on their project to foster effective communication between themselves to support each other with their career development.

BA’s anxieties about career were salient and palpable in their engagement throughout the supportive intervention. His challenges precipitated the couple’s involvement in the program. In their conversations, BA’s main goal was for EL to understand his challenges with career exploration. His uncertainty and doubt around his career were evident at the outset of their conversation, with BA starting the conversation saying, “I don’t know, like, I don’t know.” He continually tried to express his feeling of being overwhelmed and stuck throughout their conversation by overtly expressing his doubts and uncertainties, outlining his decision-making processes, and describing himself.

BA attributed his experience of feeling suffocated and overwhelmed by his career uncertainty to his familial context. In his home country, BA grew up in a highly accomplished family with family members having achieved tremendous success and influence in their respective careers. His familial background contributed to the importance he placed on his career, and his perceptions of a “worthy” or “successful” career. This meaning-making amplified the importance of BA’s career decisions resulting in him feeling stuck and challenged to authentically share his difficulties with his family.

EL experienced a complex multitude of emotions which impacted how she engaged in supporting BA. EL was concerned and worried for BA about his career challenges. From her care for him arose a sense of “need” to support him through his challenge. However, EL noted that due to being in the same position in their career development, she also experienced similar anxieties. Due to BA’s overt expression of anxiety, EL was conscious of regulating her own anxiety. Rather than sharing her anxieties, EL reported that she tried to portray herself as confident in her career development to support BA through his challenges. This action resulted in EL feeling isolated in her career development while BA felt “useless” in their partnership, thinking “they focus too much on his problems.” Her experience of these complex and incongruent emotions influenced her to disengage with BA’s emotions and provide support in the form of problem-solving by expressing her opinions, providing encouragement to engage BA in action, and describing herself and how she engages with the challenges she experiences.

As they engaged with each other, the misalignment between the emotional support BA was seeking and the instrumental support EL provided—motivated by their own emotions and action tendencies—resulted in relational ruptures. The following short exchange highlights their dynamic:

BA: The problem that I also have is that I don’t know exactly what I want to get into. That’s why my brother suggested a consulting firm, right? Because he was like, “…since you basically have no clue what you want to do, maybe a consulting firm might be interesting because you can gather some knowledge about a bunch of different fields and you don’t necessarily need to have any knowledge about them to start out with.” But yeah, it’s hard.

EL: You could also look, though.

BA: No, I know… but like, I don’t know. I’m trying to like, think about, these days, about what I want to do, right? …

During his self-confrontation BA reported feeling misunderstood, thinking that EL did not understand his challenge and was unable to help with his internal struggle. Experiencing BA’s reluctance to engage in action and follow through with the advice she provided despite their repeated dialoguing, EL experienced stress and frustration. Understanding that historically when EL provided advice, they had conflict, BA sought to regulate her frustration by moving away from conversations they are “stuck” on to avoid conflict. Being cognizant of the importance BA placed on his career decision-making and her own frustration, EL set boundaries for how involved she became with BA’s career uncertainty in order to regulate his feeling of being overwhelmed and her experience of frustration.

The one-sided misalignment in the support BA needed and EL provided became evident throughout the telephone monitoring period where they highlighted engagement in seeking support from other professionals rather than each other in their career challenges. For example, BA reported connecting with other supports such as professors and academic advisors to focus on completing his courses to address the academic challenges he faced. Motivated by her feelings of concern and care for BA, EL reported supporting BA by discussing his career opportunities, going with him to the academic advising, scheduling and planning studying for exams, and trying to motivate him. At the same time, EL highlighted that she did not want to “intrude” on his decision-making. For her own career development, perceiving that she could not seek support from BA due to his own challenges, EL reported connecting with students, program assistants, professors, and family members about post-graduate education, financial feasibility of continuing her education, and her immigration status. Notably, neither partner identified how they experienced support from the other throughout their telephone monitoring.

While their dynamic was similar in the final conversation, it provided an example of how the partners aligned in providing and receiving support. For example, in the following exchange EL spoke about her uncertainty of being admitted to graduate school:

EL: My grades right now, they’re not that competitive, they’re ok. I’m still in Honours but the cut-off grade was 75, I got, like, 75.6, because of my commerce classes, my account, stupid accounting. The worst idea ever, anyway. Honestly, such a horrible mistake.

BA: I remember…

EL: I could have done a super easy minor, like Spanish, but I didn’t.

BA: I remember M also complained so much about accounting, she’s like: “That’s the hardest class.”

Here, while EL moved the conversation away, BA’s response kept her on the topic and resulted in elaboration. BA’s empathy allowed EL to continue expressing her frustration at picking a particularly difficult class which impacted her GPA, and possible admission to graduate school. EL noted that she “values” the emotional support BA provided “a lot.”

This case study demonstrates how emotions emerge during key career development transitions. It further highlights how our engagement with others can help or hinder individuals’ processes through these periods. Anxiety about one’s future career is a particular emotion that surfaced in this dyad. EL’s anxiety generated a supportive response from BA. At the same time she directed her own anxiety about her career future to actions outside the dyad.

### TC and MP

TC and MP were roommates who came from different parts of the same country. They did not know each other before becoming roommates in Canada. Both were in their final year of graduate degrees. Their project involved supporting each other in exploring their interpersonal and intrapersonal experiences related to career development as they prepared to transition from university to their professional lives. TC and MP provide another example of how emotions can influence the ways in which individuals assign meaning to their vocational experiences during their transition to emerging adulthood in a new country.

TC and MP worked together on their project to support each other through a collaborative exploration of their intrapersonal and interpersonal experiences relevant to their career development. As they pursued their joint project, they shared feelings of appreciation toward each other and emotions of fear, doubt, and disappointment. They also had experiences of hope as they regained a sense of self-confidence about the uncertainty they felt about their career transitions.

During their initial conversation, the main joint goal of MP and TC was to support each other emotionally and practically through moments of uncertainty. A key aspect of their relational dynamic at this time was the encouragement and support they provided one another. For example, at one point in their conversation, TC shared her disappointment of not having the work experience she would have liked to have had at this point. MP responded with a supportive statement that let TC know that she was hearing her and understanding her, while also encouraging her by saying “I know. But it’ll happen. I, I have a feeling. Something will arrive.”

A focus of TC and MP’s initial conversation involved sharing their challenges, experiences, and perspectives about job searching. They shared their anxiety related to the uncertainty of how the job searching process worked in Canada. A worry for MP was realizing that there were cultural differences between Canada and her home country regarding effective strategies for job searching, such as the best ways to structure resumés and cover letters. For example, at one point, MP described to TC the pressure that she had been feeling and shared, “I’ve just been trying to get that correct.” TC responded to MP by acknowledging her experience of uncertainty to validate her experience. TC then proceeded to add her own perspective and gave MP advice that one of the most important aspects of job searching in Canada is networking. As they further explored the role of networking in gaining employment, MP expressed her worry and fear that networking would require her to step outside her comfort zone. TC again validated MP’s fear and encouraged her by recalling a situation in which TC bravely stepped outside her comfort zone in a networking event. In this part of the joint action, TC’s goal was to remind MP of her existing courage while at the same time normalizing and validating her experience. TC further encouraged MP by inviting her to reflect on how rewarding this experience was for her as she obtained a professional recommendation from this networking event. During this interaction, the emotional vulnerability that MP displayed enabled TC to gain a deeper understanding of her overall experience of uncertainty around job searching. TC was then able to create space for MP by validating her emotional experience while at the same time offering more instrumental support to address the specific areas of worry that MP had raised.

TC and MP also supported each other in their anxieties related to transitioning out of university. Both TC and MP shared feelings of comfort in being understood in their unique, yet relatable experiences. At one point in their conversation, MP shared her disappointment in some advice she had been given by people regarding building her professional identity. For example, she said “some of the advice I got is like ‘oh, you should travel the world, you know, find yourself.’ I don’t have the luxury to do that. A lot of people (yeah) suggested that. In fact, you’d be surprised.” TC responded with a validating statement and elaborating on her own shared anxieties about financial stability as they transition into their professional lives. MP also shared her fear of not having previous work experience during this transition, “See, that’s my problem, right? I don’t know like these small, small things that are work related, because I have no experience in the real world, working (yeah). Small things like contracts, negotiating for salary, how do you do that, you know? How do you ask someone for money?”

Throughout their engagement in their joint project, TC and MP were also vulnerable with each other during moments of relational misattunement. During one of their conversations, TC noticed MP’s experience of self-doubt and challenged some behaviors and cognitions associated with MP’s insecurities. MP responded to this challenge by verbalizing that she felt misunderstood. Communicating with TC involved preparing for the possibility of rejection. It felt helpful to MP as a way of coping with the fear of uncertainty. MP and TC’s self-disclosures during the self-confrontation phases gave us a deeper appreciation of their intrapsychic processes related to this particular part of their conversation. For example, during the self-confrontation, TC shared that she understood MP’s need to have a space to vent about her insecurities and that she believed it would be helpful to also challenge her gently. Meanwhile, in her self-confrontation, MP shared that she felt stuck in her current way of thinking and that she anticipated what TC might say to her in response to her experience of self-doubt. Thus, in this way, the role of emotion also included enabling meaningful discussions and facilitated opportunities for gaining a deeper understanding of each other’s perspectives and experiences in their own journey of transitioning to their professional lives post-graduation.

Another joint goal related to TC and MP’s joint project was to explore the possibility of relocating for work. They both shared a feeling of uncertainty in exploring this option. For TC, this uncertainty was accompanied by an open curiosity about what it might be like to live in a different city in the pursuit of career opportunities. While open to this option, TC also expressed feeling appreciated for having the support of her family members who live in the current city. MP, on the other hand, felt a more pronounced fear about leaving the comforts of her current city to pursue potential career options that still seemed highly uncertain. For example, she shared with TC:

... [Current city] is like a comfort zone now, right? (yeah) We’ve been here for eight months, we know people here, we’re used to the system, it’s starting to feel like home, yeah (yeah). And, but, what if this is not it? Like, what if this is not where our opportunity is? Then we’ll have to look (yeah) far and wide, although it scares me. I can’t (I don’t want) think about, yeah, not living with you or even going to the entirety, like a new city where I don’t know anyone, and that terrifies me.

During the latter phases of their involvement in the supportive program, TC and MP’s joint goals became more focused on supporting each other by sharing their own experiences of career-related skills that they had been developing since their last conversation together. By supporting each other in this way, they expressed feelings of deep appreciation for each other and increased feelings of confidence and hope regarding their career trajectories. For example, TC shared how she felt inspired by MP’s approach to networking and how it encouraged her to take initiative. This reciprocity is demonstrated in the following exchange:

TC: Um, I think back to it, for me, I wasn’t really talking to a lot of people, and I think when I saw you progress in that way, I also started reaching out to people that I (yeah) could potentially see either being, ah, mentors or yeah, friendly to people who could be (mh-hum). It’s not as big a turnout as I expected, but it’s still going somewhere.

MP: Yeah. You won’t expect results immediately, but if you start now (yeah), in the future then you can, you can go back and ask them and say: “Can you help me with this?” It wouldn’t be as awkward as what I felt, because I was reaching out and immediately asking for help, which is not that nice. But if you start earlier, and you build that relationship, it wouldn’t be as hard to ask them (yeah) for a favor or to give you a reference. Now I’m just asking people, like, straight, shamelessly asking them: “Oh, can you give me a reference?” or “Can you help me with my application?” And they’re nice, they’re very positive about it, but it would be better to (inaudible) for sure. And what else can we discuss in our session that has changed now?

TC and MP also shared their appreciation for having created a joint friendship that felt trustworthy and supportive. TC shared her appreciation for MP by saying:

Yeah. Yeah, it’s always nice to know that there is someone you can completely trust with certain things, and, like, I think we we’ve spoken about this before, it helps a lot that you’re not in my stream (yeah) because you’re not my competitor in any way. Like all, with all my friends, like with [other friends], they’re very helpful, they’re very sweet, but things like interview and job search is not something that can be discussed, because they are competitors and (yeah) and you want to reduce that competition as much as possible, so (yeah), I mean, that’s how it works, right? So just knowing that there’s someone, like, who I can completely trust with certain things, that’s really helpful. Like even things like my work permit or, like, sensitive information that I normally wouldn’t show others, I can share that with you (inaudible) interest.

Throughout their participation in their project, TC and MP were collaborative and highly engaged with each other. Both demonstrated a level of trust and vulnerability in their relationship as they shared their perspectives, fears, and hopes regarding finding a job after graduation. Having the openness and capacity to share with each other about their emotional and cognitive experiences illustrated the strong relational foundation that TC and MP had built with each other. The reciprocal sharing and understanding of each other’s emotional experiences facilitated a deeper understanding of the kind of support that each person needed at a given time.

## Discussion

The aim of the presentation of these cases was to illustrate how emotions related to transition in the career development process are present in the joint actions of young adults. These two cases show that the transitions reported here address significant career development issues (e.g., Meerkins et al., 2021). Furthermore, they reveal how emotions are an integral and critical part of the projects engaged in by the participants. These cases are representative of the co-action of acculturative and development processes that young adults in a new country face (Juang & Syed, 2019). Both cases illustrate that the participants’ anxiety and uncertainty about their future careers were contextualized by them because they were in a new country. The current description also highlights that career and other transition-related challenges faced by individuals are constructed and acted on in social and relational environments, which themselves have emotional dimensions. In contrast to the cases illustrated, Autin and colleagues (2018) reported that those undocumented students in the United States experienced emotions such as anger, shame, depression, and frustration. However, these emotions were addressed by the social support they received, which included its inherent emotional dimension. Both cases reported here illustrate social support as a major factor in the participants’ expected educational and/or vocational adaptation. This view supports the contention that understanding how emotions operate in goal attainment requires an understanding of the social (relational) context (English & Growney, 2021). Contextual action theory suggests that career goal attainment is heavily influenced by the relational and social context (Young & Valach, 2019), which is evident in these cases. The cases also reflect the shift away from individualist explanations of vocational behavior (e.g., Blustein et al., 2008). The CAT perspective clarifies how progress is made or lost in career projects. The cases presented here serve to illustrate that emotional attunement promotes progress toward goals even in the absence of specific behavioral goal setting. Furthermore, the cases illustrate Rimé and colleagues’s view (2020) of the importance of both the relationship of emotion to goal pursuit and the social sharing of emotion.

 A major emotion in these dyads involved the expression of anxiety about obtaining work after graduating from university. Anxiety has been a consistent factor in career research, particularly for young people entering the world of work (e.g., Leong, 1985; Xu, 2024). Anxiety in the illustrated dyads was about specific obstacles to obtaining employment and realizing their career goals in a new country and culture. Each participant had different approaches to the challenges they shared with their dyad partner. However, attempts at providing interpersonal support were not always successful. In addition, the dyads engaged in strategies to portray confidence and optimism about the future.

Emotions played a major role in these joint actions. At every step, the participants’ emotional states served to direct the conversations and the strategies they employed (Young et al., 1997). Cognitions were associated with the emotional states. They both influenced and were influenced by the accompanying emotions. There appeared to be a reciprocal or parallel development between the members of each dyad. They were also involved with others outside the dyad in the pursuit of their career projects.

 In one dyad, the differing approaches of each participant served as examples of what was possible, while in the other dyad, the differing approaches elicited a negative emotional response and relational disruption. Reflective of a process suggested by Frijda (1986), BA’s cognitive evaluation of EL’s suggestions as impractical was a factor in BA’s emotional response. Thus, the emotional response in the context of the relationship was also a component of the cognitive appraisal of the suggestions. The relationship context was very different in the BA-EL dyad, as they were a couple, whereas the second dyad were roommates, which may account, at least in part, for the different emotional responses (Young et al., 1997). The TC-MP dyad was able to move forward due to a firm foundation of trust in the context of their personal relationship, which they noted was not affected by direct competition. Both dyads had times when one partner was “stuck.” The TC-MP dyad was able to move past this barrier with expressions of understanding and reassurance. The BA-EL dyad eventually made progress toward relational repair through BA’s expression of understanding of the challenges EL faced. This example illustrates the point that it was the relationship itself that needed repair rather than resolution of the specific issue that caused the rupture in the relationship (Graham & Bitten, 2015).

 Emotions motivated the joint conversations (Barrett, 2017; Young et al., 1997, 2002). They also played an important role in the participants’ choice of strategies in the conversations. For example, the participants self-regulated by engaging in the conversations about difficult and anxiety-provoking topics. By discussing their emotions and challenges, they seemed able to lower their anxiety (Greenberg et al., 1993).

In the EL-BA dyad, EL regulated her emotions when she realized they were “stuck” on a topic and she then refrained from further discussion about the topic, which may not have been immediately helpful in making progress on BA’s career project. As noted by English and Growney (2019), an emotional regulation strategy can work against longer term goal attainment when emotion regulation intended to decrease negative affect creates unwanted distance in a relationship. Thus, the dyad’s joint actions illustrate that emotions may not always promote progress, depending on relational processes.

EL may have misunderstood an underlying emotional state by focusing on the content of BA’s verbal communication and not addressing his insecurities and feelings of inadequacy stemming from issues related to his family of origin. Thus, BA’s emotional state can be seen as impeding his career and personal development in the context of the relational process. In contrast, MP and TC, who faced similar emotional challenges, appeared to more easily move past these challenges through expressions of understanding, reassurance, and appreciation. From this example, one can see that it is important to attend to emotional states, especially underlying emotions and associated cognitions (Greenberg, 1993), and that the relational process is critical in how emotions function to promote or impede progress toward goals. It is interesting to note that very little formal problem-solving, or goal setting was evident in the joint actions. Rather, interpersonal support and explaining one’s own approach to circumventing obstacles seemed to spur action outside the dyad’s joint action. In other words, the understanding and support offered in the joint action, which can be seen as attention to the emotional states of each person, appeared to be the main ingredient necessary for progress. Further, it is evident in these cases that emotional attunement promoted progress toward career goals, while relational disruption impeded it. As noted by Young et al. (1997), each member of a dyad might share an overarching goal, their emotional engagement can contribute to difficulties in the relationship through differing constructions of the career project. This aspect of the relational process can reach resolution or acceptance through dialogue.

### Implications for Practice

From the practice perspective, three specific implications can be drawn from the current illustrations. First, these cases provide additional support for the need for counselors to appreciate and attend to client emotions as present and critical in career development actions and projects. Moreover, the illustrations suggest that emotion is present in complex ways within these projects as they involve the co-action of developmental, acculturative, and other transitions. They are also socially based. Career counselors are invited to consider the emotional dimensions of the goal-directed actions in which the client is engaged, particularly those actions that may be problematic for the client. They may also be able to attend to the emotional regulation in the specific career-related actions of their clients. For the mid-term project, counselors can ask whether the project is emotionally functional for the client. Career counseling is much more than helping a client to determine a suitable occupation. Client emotion in relational contexts needs to be considered in counseling interventions.

Second, the evidence of emotions in the actions in dyadic processes invites counselors to consider how they can address emotions specifically in counseling. Identifying and talking through emotions are established methods in counseling generally and can be applied to emotional processes in career counseling (e.g., Strepparava et al., 2017; Watson, 2015). One straightforward method is to invite clients to recount pertinent conversations with others and in doing so access emotional processes. More specifically, contextual action theory has identified and described five primary tasks of counseling in which emotion is well integrated. These tasks are creating and maintaining a working alliance, identifying actions, projects, and careers, addressing problematic actions and projects, addressing emotions and emotional memory, and connecting with daily life (Young, Valach, & Domene, 2015). These tasks are not listed sequentially and thus critical in throughout the counseling process. Even though CAT does not preclude the use of any specific procedure or intervention, it is clear that emotion in relational contexts needs to be considered in career counseling interventions.

Third, the procedure used in the cases presented here provides an opportunity for practitioners to attend to the emotional experiences embedded in the joint actions of clients with others (Young et al., 2022). This procedure can access and support the joint actions between couples, friends, family members, and others that occur in daily life. These are places where emotions related to transition and career are present and intimately connected with the action that in occurring. The method used in these illustrations departs from conventional career counseling. Rather than assessing vocational preferences or aptitudes or setting specific goals, the actions and relationships with others are seen as influential in the setting of goals and progress toward those goals (Young et al., 2002). The relational focus of the CAT-informed counseling intervention described above aligns with meta-analytic literature that has identified individualized interpretations and feedback and attention to building support as key ingredients of effective career interventions (Brown & Krane, 2000). In particular, the video playback allows clients access to the emotional aspects of their actions. The written narrative feedback portion of the intervention provides clients with opportunities to receive explicit feedback about the actions in which they are engaging in relation to their specified goals.

### Limitations

The two cases reported here are unique and cannot be applied directly to other cases. The counselors involved in these dyads also served as research assistants on this study. The intervention method and their role as counselors were highly prescribed beforehand, as outlined in the method described above. They were also involved with the broader research team in the data analysis, which allowed increased awareness of the emotional components. Finally, the role of emotion was the focus of the illustrations presented here. While emotion is best understood in the fullness of both context and action, it is acknowledged that some aspects of each case are not addressed in detail.

## Conclusion

The cases described in this paper are important in several ways. First, they identify and describe two joint transition-related projects in which young adults are engaged. These cases illustrate how people can be engaged with each other in career and other transition-related actions. Career does not reside solely within the individual. It is jointly constructed. Second, these joint projects, particularly as they contribute to long-term career, include important emotional components. These cases speak to approaches to career development and related interventions that recognize the primacy to emotion.
